# Increased Antimicrobial Activity of Colistin in Combination With Gamithromycin Against *Pasteurella multocida* in a Neutropenic Murine Lung Infection Model

**DOI:** 10.3389/fmicb.2020.511356

**Published:** 2020-09-22

**Authors:** Yanqin Li, Mengjuan Xie, Junwen Zhou, Hao Lin, Tianan Xiao, Liqin Wu, Huanzhong Ding, Binghu Fang

**Affiliations:** ^1^National Risk Assessment Laboratory for Antimicrobial Resistance of Animal Original Bacteria, South China Agricultural University, Guangzhou, China; ^2^Guangdong Provincial Key Laboratory of Veterinary Pharmaceutics Development and Safety Evaluation, South China Agricultural University, Guangzhou, China; ^3^Guangdong Center for Agricultural Products Quality and Safety, Guangzhou, China

**Keywords:** colistin, gamithromycin, combination, therapy, *Pasteurella multocida*

## Abstract

We investigate the antimicrobial activity of combined colistin and gamithromycin against nine *Pasteurella multocida* strains by testing *in vitro* susceptibility. Two high-colistin minimal inhibitory concentration (MIC) isolates (D18 and T5) and one low-colistin MIC isolate (WJ11) were used in time-kill tests and therapeutic effect experiments using a neutropenic murine pneumonia model over 24 h. Pharmacokinetics (PK) in plasma was calculated along with pharmacodynamics (PD) to determine the PK/PD index. Synergy between colistin and gamithromycin was observed using high-colistin MIC isolates, equating to a 128- or 256-fold and 4- or 8-fold reduction in colistin and gamithromycin concentration, respectively. Interestingly, no synergistic effect of the combination on low-colistin MIC isolates was observed. However, regardless of the MIC difference among isolates, each drug tended to reach the same concentration in all isolates subjected to combined treatments, which was verified by the time-kill tests presenting similar rates and extent of killing for isolates D18, T5, and WJ11. The AUC_(__0__–__24 h)_/MIC index was used to evaluate the relationship between PK and PD, and the correlation was >0.89. The relevant gamithromycin doses for combined therapy were determined, and the value decreased from 6- to 35-fold compared with monotherapy. Combined colistin and gamithromycin therapy provides a more potent therapeutic regimen than monotherapy against *P. multocida* strains.

## Introduction

*Pasteurella multocida* is believed to be a causative factor in many host diseases, including fowl cholera, porcine pneumonic, and bovine hemorrhagic sepsis, all of which result in significant economic losses. *P. multocida* strains are classified into five serotypes (A, B, D, E, and F) based on capsule antigens or 16 serotypes (L1–16) according to differences in lipopolysaccharides (Heddleston et al., 1972; Marina et al., 2006). It is often difficult to select a suitable vaccine to protect hosts from infection due to the large number of serotypes and capsular serotypes of *P. multocida*. Therefore, timely and effective implementation of antibiotics remains crucial.

Bovine respiratory disease (BRD) associated with *Mannheimia haemolytica*, *P. multocida*, and *Histophilus somni* is one of the most economically important diseases that occur in feedlots, and the global losses are estimated at over $3 billion per year (Watts and Sweeney, 2010). Gamithromycin is a 15-membered semisynthetic macrolide antibiotic that has been approved for treatment and prevention of BRD and swine respiratory disease (SRD) caused by *Actinobacillus pleuropneumoniae*, *P. multocida*, and *Haemophilus parasuis* (European Medicines Agency [EMA], 2008, 2014, 2015). Excellent pharmacokinetic (PK) profiles have been reported for gamithromycin in cattle, pig, foal, and broiler chickens, characterized by fast and complete absorption and distinct tissue penetration (Steeve et al., 2011; Watteyn et al., 2013; Wyns et al., 2014; Berlin et al., 2017). Compared with other second-generation macrolides, gamithromycin might be advantageous for the treatment of bacterial respiratory diseases (Rossi et al., 2010; Wyns et al., 2014). However, the similar chemical properties and mechanism of action between gamithromycin and other macrolides leads to the same pattern of drug resistance. Research on macrolides against *P. multocida* strains demonstrates that *erm*(E) can induce serious resistance against gamithromycin, tulathromycin, tylosin, and tilmicosin in *P. multocida* at MICs ≥512 mg/L (Benoit et al., 2011). Similarly, the combination of *erm*(42) and *msr*(E) – *mph*(E) genes results in a significant increase in the MICs of gamithromycin and tildipirosin (Michael et al., 2012). It is, therefore, necessary to combine gamithromycin with other potential drugs to reduce the risk of resistance and extend the useful therapeutic life of gamithromycin.

Colistin is considered a drug of last resort for preventing Gram-negative bacterial infections, and it has been approved for treatment of relevant diseases in food-producing and companion animals (European Medicines Agency [EMA], 2019). However, the positive therapeutic effect must be weighed against potential kidney damage (Nation et al., 2014; Omrani et al., 2015). Therefore, colistin-based combination therapies have received increasing attention in recent years, and the focus has been to improve the antimicrobial activity of each drug while reducing the dose-dependent toxicity of colistin.

The purpose of this study is to provide an alternative and more effective therapeutic regimen for infections caused by *P. multocida* while reducing the dose-dependent side effects of colistin and extending the therapeutic life of gamithromycin, which is the newest macrolide drug. Several experiments, including *in vitro* susceptibility and time-kill tests and *in vivo* PK and pharmacodynamics (PDs) assays were carried out using a neutropenic murine lung infection model.

## Materials and Methods

### Bacterial Strains

Nine isolates of *P. multocida*, including a standard strain (CVCC444) obtained from the China Institute of Veterinary Drug Control and eight clinical strains isolated from the livers of dead chickens (WJ11, WW18, WN17, D18, T5, and XX12) and pigs (PmP2 and PmP17) were used in this work. A multiplex PCR assay for capsular serogroup identification was conducted based on a previously reported method (Townsend et al., 2001). Whole-genome sequencing was conducted to identify the resistance-associated genes by Novogene Bioinformatics Institute (Guangzhou, China).

### Antibiotics and Media

Colistin sulfate powder was purchased from Sigma-Aldrich, and gamithromycin powder was obtained from Quality Control Chemicals Inc. (Walnut, CA, United States). Cefquinome was purchased from Qilu Animal Health Products Co., Ltd. (Jinan, China). Enrofloxacin, tilmicosin, tylosin tartrate, and florfenicol were obtained from the China Institute of Veterinary Drug Control (Beijing, China). Antibiotic stock solutions were prepared according to Clinical and Laboratory Standards Institute (CLSI) criteria before testing (Clinical, and Laboratory Standards Institute (CLSI), 2018). Mueller-Hinton agar was obtained from Qingdao Hope Bio-Technology Co., Ltd. (Qingdao, China). Sterile defibrinated sheep blood was purchased from Guangzhou Ruite Biotechnology Co., Ltd. (Guangzhou, China). Mueller-Hinton II broth (25.0 mg/L Ca^2+^ and 12.5 mg/L Mg^2+^; Becton, Dickinson and Company, Sparks, MD, United States) was used in all experiments.

### Antimicrobial Combination Susceptibility Testing

The susceptibility of the selected *P. multocida* isolates and the quality control isolate (ATCC 25922) to colistin, gamithromycin, cefquinome, enrofloxacin, tilmicosin, tylosin tartrate, and florfenicol alone was determined using the microdilution method in broth (conducted in triplicate) according to the guiding principles of the CLSI. Based on the action mechanism of each drug, the antimicrobial activity of colistin or cefquinome (drug A) in combination with gamithromycin, enrofloxacin, tilmicosin, tylosin tartrate, or florfenicol (drug B) was determined with the checkerboard method, according to the minimal inhibitory concentration (MIC) of each drug. Briefly, 50 μL of MH II broth was added to each well, and 50 μL of the indicated concentration of colistin or cefquinome was added into the first well of each line. This was followed by multiproportion dilution. Next, 50-μL samples of different concentrations of gamithromycin or the other drugs mentioned above were added to the dilution medium, and a 100 μL inoculum with a cell density of ∼1 × 10^6^ colony-forming units (CFU)/mL was added to each well. The plates were then incubated at 37°C for 24 h. According to the EUCAST definitive document E.Def 1.2 (Mccullough, 2010), the fractional inhibitory concentration (FIC) index for combined administrations was defined as the sum of the FIC of drug A and the FIC of drug B, where FIC is the MIC of the drug combined with other drugs, divided by the MIC of the drug alone.

### Time-Kill Assays

*In vitro* time-kill assays were used to evaluate the effects of colistin alone and in combination with gamithromycin over 24 h. Three isolates of *P. multocida* (D18, T5, and WJ11) were tested. A *P. multocida* inoculum at ∼5 × 10^6^ CFU/mL was treated with colistin alone at 0.25, 0.5, and 1 mg/L; gamithromycin alone at 0.125, 0.25, and 0.5 mg/L; and with colistin in combination with gamithromycin. At 0, 1, 2, 4, 6, 8, 12, and 24 h after incubation, 100-μL samples were taken from each tube for colony counting. A growth control without drug administration was included in all tests.

### Mutant Prevention Concentration

The tested bacterial strains (D18, T5, and WJ11) were incubated in MH II broth for 12 h. The bacteria-containing broth was centrifuged at 3500 *g* for 15 min and resuspended in fresh MH II broth to obtain a solution containing 3 × 10^10^ CFU/mL of *P. multocida*. The agar plates were produced by adding different concentrations of colistin and gamithromycin alone or in combination into the Mueller-Hinton agar medium supplemented with sterile defibrinated sheep blood. Aliquots of 100-μL inoculum were plated on four agar plates containing the same concentration of drug(s). Mutant prevention concentration (MPC) was defined as the lowest concentration that prevented the growth of bacteria after 72 h of incubation at 37°C.

### Analysis of *in vitro* Postantibiotic Effect

For determination of the postantibiotic effect (PAE) of colistin, gamithromycin was tested on D18, T5, and WJ11 isolates alone and in combination with colistin, and a 1 × 10^8^ CFU/mL inoculum of each isolate was used after overnight incubation in MH II broth. A 0.5-mL sample of the inoculum and 0.5 mL of colistin or gamithromycin alone at 1 × or 4 × MIC, was added to 4 mL of fresh broth and incubated in a 37°C water bath for 1 h. Meanwhile, the same operation was performed for combinations of colistin at 0.25–0.5 mg/L and gamithromycin at 0.125–0.25 mg/L. Controls without drugs were included. Each inoculum was diluted 1000-fold after incubation for 1 h, and this was the starting point for PAE testing (0 h). Aliquots of 0.1 mL were withdrawn to count the bacterial burden as described above in time-kill tests at 0, 1, 2, 4, and 6 h. PAE was calculated using the equation: PAE = T – C, where T and C represent the time taken for drug-exposed (T) and control (C) cultures to increase by 1 log_10_ after drugs were removed (Mackenzie and Gould, 1993).

### Murine Pneumonia Model

Five-week-old specific-pathogen-free female Institute of Cancer Research (ICR) mice weighing 20–22 g obtained from the Laboratory Animal Center of the Southern Medical University (SCXK 2016-0041, Guangzhou) were used in this experiment. Animals were maintained in the Laboratory Animal Center of the South China Agricultural University (SYXK 2014-0136, Guangzhou) in accordance with the National Standards for Laboratory Animals in China (GB 14925–2010). Neutropenia was generated by intraperitoneal injection of cyclophosphamide [TCI (Shanghai) Development Co., Ltd., Shanghai, China) 4 days before the 150-mg/kg dose and 1 day before the 100-mg/kg dose prior to lung infection (Vogelman et al., 1988). Three strains, namely, D18, T5, and WJ11, were incubated in MH II broth. Experimental mice were anesthetized via inhalation of isoflurane and then were hung on an adjustable-angled bracket by hooking their teeth. Endotracheal intubation was performed by importing 50 μL of a logarithmic-phase bacterial suspension (10^8^–10^9^ CFU/mL) into the lung through the larynges rimae using a preinserted tracheal tube (Zhou et al., 2018). All experimental protocols were approved by the Committee on the Ethics of Animals of South China Agricultural University.

### PK Analysis in Mice

Pharmacokinetics analysis of colistin (2.5 and 5 mg/kg), gamithromycin (1.5, 3, 6, 12, and 24 mg/kg) alone and in combination (2.5 mg/kg colistin, 3 mg/kg gamithromycin) was performed in 176 neutropenic lung-infected ICR mice following subcutaneous administration (SC). Plasma and lung tissue samples were withdrawn at the specified time point (four mice at each time point).

The concentration of colistin and gamithromycin in plasma and lung tissue was measured using a previously reported method (Ma et al., 2008; Huang et al., 2010; Fu et al., 2018). The limit of quantification for both colistin and gamithromycin was 0.01 mg/L in plasma and 0.2 μg/g in lung tissue. Recovery of colistin and gamithromycin in plasma and lung ranged between 88.2 and 113.1%. All inter- and intra-assay coefficients of variation were <10%. PK parameters in plasma and lung samples were estimated using a non-compartmental analysis method with WinNonlin software version 5.2.1 (Pharsight, St Louis, MO, United States).

*In vitro* plasma protein-binding experiments for gamithromycin were conducted by adopting an equilibrium dialysis method for 72 h at 4°C. The corresponding unbound plasma PK parameters were obtained and used for the integration of PK and PD data.

### Analysis of PD in Mice

Pharmacodynamics analysis of combined colistin and gamithromycin treatment in 264 lung-infected neutropenic mice was performed at 2 h after administration of the inoculum. To this end, 2.5 or 5 mg/kg colistin was subcutaneously administered every 12 h in combination with 1.5, 3, 6, 12, or 24 mg/kg gamithromycin, and colistin and gamithromycin were also tested alone at 1.5, 3, 6, 12, 24, 48, and 96 mg/kg (*n* = 4 for each group). Animals were humanely sacrificed 24 h after drug administration. Lung tissues were then removed under sterile conditions with the help of scalpels on a clean bench and homogenized in 0.9% sterile saline water with a tissue lyser. An appropriate diluent of homogenate was plated on Mueller-Hinton agar supplemented with sterile defibrinated sheep blood. Control animals without any drug administration were sacrificed at the beginning (0 h) and end (24 h) of therapy.

### Analysis of the PK/PD Relationship

The relationship between the *in vivo* antimicrobial activity of gamithromycin alone or in combination with colistin and the ratio of plasma PK parameters to the MIC of gamithromycin was assessed. An inhibitory effect E_max_ model from the Phoenix WinNonlin identification module was used for analysis based on the equation: E = E_max_ − (E_max_−E_0_) × C_e_/(C_e_ + EC_50_), where E is the antimicrobial effect (i.e., the change in bacterial burden in CFU per lung), E_max_ is the change in log_10_ CFU/lung for control groups, E_0_ is the maximum effect, C_e_ is the PK/PD index under analysis, and EC_50_ is the C_e_ value at 50% of E_0_.

### Statistical Analyses

The statistical significance of the difference between results was analyzed with a *t*-test provided by SPSS statistical software.

## Results

### Drug Screening

Many types of drugs, such as β-lactams, macrolides, and fluoroquinolones, are known to be effective against *P. multocida* and are often used to treat infections caused by this pathogen. We determined the MICs of frequently used drugs in the veterinary clinic, including cefquinome, enrofloxacin, tilmicosin, tylosin, and florfenicol, against three strains (D18, T5, and WJ11). With the exception of the MICs of tilmicosin and florfenicol (2–4 mg/L), the MICs of the other drugs against the three strains were more than 64 mg/L. Based on the action mechanism of these drugs, the FIC index was also calculated for the combination of cefquinome and gamithromycin. Although cefquinome and gamithromycin were found to have a synergistic effect, the concentration of cefquinome in the combination administration still remained high at 8–16 mg/L, which is higher than the clinically available drug concentrations. Therefore, the combined effect of colistin and gamithromycin against *P. multocida* was further investigated.

### *In vitro* Susceptibility Testing and Time-Kill Assays

Minimal inhibitory concentrations of colistin and gamithromycin alone and in combination against the nine isolates in MH II broth are listed in Table 1. MICs of colistin against CVCC444, WJ11, WW18, WN17, D18, T5, XX12, PmP2, and PmP17 ranged from 0.5 to 32 mg/L, and the gamithromycin MICs for all the strains were 0.5 mg/L. A synergistic effect was observed in the form of a reduction in colistin MICs by 128- to 256-fold following supplementation of gamithromycin against high-colistin MIC isolates (D18, T5, XX12, PmP2, and PmP17; hereafter referred to as R isolates). In addition, the combination of 0.125 mg/L colistin and 0.125 mg/L gamithromycin appeared to be optimal for the R strains. Interestingly, for the lower colistin MIC strains (CVCC444, WJ11, WW18, and WN17; hereafter referred to as S isolates), there was no such effect, but an additive effect was observed in antimicrobial susceptibility tests of combination treatments.

**TABLE 1:** Serotype summary for each *Pasteurella multocida* strain, MICs of colistin alone or in combination with gamithromycin, and fractional inhibitory concentration index (FICI) of the combinations against *Pasteurella multocida* strains in Mueller-Hinton broth.

*Pasteurella multocida* strain	Characteristic	MIC (mg/L)				
		CST	GAM	CST (in combination)	GAM (in combination)	FICI
CVCC 444	A	0.5	0.5	0.125	0.25	0.75
WJ11	A; *mef*(B)	1	0.5	0.125	0.25	0.625
WW81	A; *mef*(B)	2	0.5	0.5	0.25	0.75
WN71	A; *mef*(B)	2	0.5	0.0625	0.25	0.53
D18	A	32	0.5	0.25	0.0625	0.13
T5	A	32	0.5	0.125	0.125	0.25
XX12	A	32	0.5	0.125	0.0625	0.13
PmP2 (pig)	A	32	0.5	0.125	0.125	0.25
PmP17 (pig)	A	16	0.5	0.125	0.0625	0.13

*CST, colistin; GAM, gamithromycin.*

**FIGURE 1:**
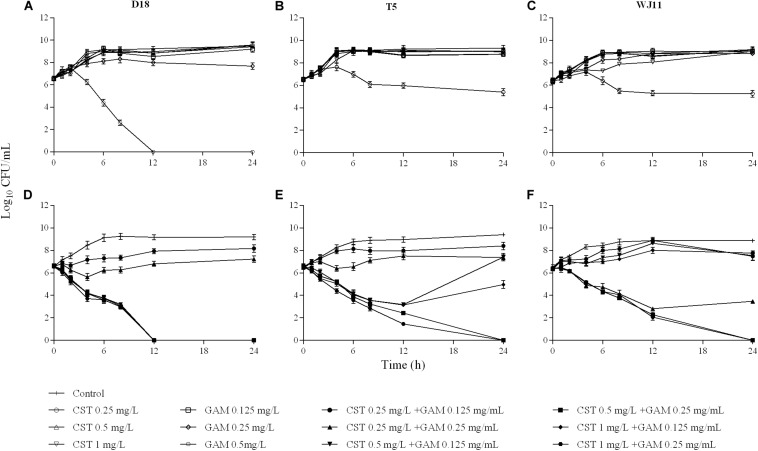
*In vitro* activity of colistin and gamithromycin alone **(A–C)** and in combination **(D–F)** against the *Pasteurella multocida* strains D18 (left), T5 (middle), and WJ11 (right). A growth control without drug administration was included in all the tests.

For all strains, all the clinically available concentrations of colistin (0.25–1 mg/L) were incapable of controlling bacterial growth (Figures 1A–C). However, the addition of 0.5 or 1 mg/L colistin to 1/4 or 1/2 MIC of gamithromycin improved the individual antimicrobial activity of both drugs (Figures 1D–F). As observed in sensitivity tests, the drug concentration tended to reach a plateau in combinations regardless of the MIC of each drug alone in isolation. Additionally, the antimicrobial activity of the combinations was not enhanced by increasing the concentrations of colistin or gamithromycin when the antimicrobial effect achieved a certain level.

### MPC and PAE

The MPCs of colistin against the strains D18, T5, and WJ11 were in the range of 64–128 mg/L, and the MPC of gamithromycin was 1 mg/L for all the strains. In contrast, the combination of colistin and gamithromycin exhibited excellent ability in blocking the growth of first-step resistant mutants of *P. multocida* when the concentrations of colistin and gamithromycin were 1 and 0.25 mg/L, respectively (Table 2).

**TABLE 2:** Mutation prevention concentration (MPC) of colistin and gamithromycin alone or in combination against *Pasteurella multocida* strains.

*Pasteurella multocida* strain	MPC (mg/L)		
	CST	GAM	CST + GAM
D18	128	1	1 + 0.25
T5	128	1	1 + 0.25
WJ11	64	1	1 + 0.25

*CST, colistin; GAM, gamithromycin.*

Postantibiotic effect values against D18, T5, and WJ11 isolates for 1 × MIC colistin (32, 32, and 1 mg/L) and gamithromycin (0.5 mg/L for all isolates) ranged from 0.29 to 0.98 h and 0.21 to 0.59 h, respectively (Table 3). Meanwhile, an increased concentration (4 × MIC) prolonged the PAE value of colistin and gamithromycin to 2.34–3.79 h and 0.51–1.83 h, respectively. Notably, 0.5 mg/L colistin plus 0.25 mg/L gamithromycin yielded much longer PAEs (2.80–3.12 h) than that achieved with 1 × MIC colistin or 4 × MIC gamithromycin, but it was roughly the same as 4 × MIC colistin.

**TABLE 3:** *In vitro* post antibiotic effect (PAE) of colistin or gamithromycin alone and in combination on *Pasteurella multocida* strains.

*Pasteurella multocida* strain	PAE (h)							
	CST-MIC^a^	CST-4MIC	GAM-MIC^b^	GAM-4MIC	0.25CST + 0.125GAM	0.25CST + 0.25GAM	0.5CST + 0.125GAM	0.5CST + 0.25GAM^c^*
D18	0.98	3.79	0.21	0.51	0.19	0.23	1.36	2.80
T5	0.29	2.83	0.54	1.83	0.45	0.84	1.66	3.04
WJ11	0.96	2.34	0.59	1.16	0.70	1.89	2.70	3.12
Mean ± SD	0.74 ± 0.39	2.99 ± 0.74	0.45 ± 0.21	1.17 ± 0.66	0.45 ± 0.26	0.99 ± 0.84	1.91 ± 0.70	2.99 ± 0.17

CST, colistin; GAM, gamithromycin. ^*a*^Colistin MICs against strains D18, T5, and WJ11 were 32, 32, and 1 mg/L, respectively. ^*b*^Gamithromycin MIC for all the strains was 0.5 mg/L. ^*c*^0.5 mg/L colistin in combination with 0.25 mg/L gamithromycin, this applies to the same presentation. *P < 0.05 for PAE of the 0.5 mg/L colistin and 0.25 mg/L gamithromycin combination versus colistin or gamithromycin alone at 1 × MIC.

### *In vivo* Pharmacokinetics

In total plasma, the maximum concentration (C_max_) of gamithromycin at 1.5, 3, 6, 12, and 24 mg/kg body weight after SC was 0.11–2.52 mg/L (mean value) within 0.25–0.75 h, consistent with 2.5 and 5 mg/kg body weight for colistin. The area under the concentration-time curve (AUC_last_) of colistin and gamithromycin was 3.51–10.52 mg⋅h/L and 0.26–5.06 mg⋅h/L, respectively (Table 4). A good linearity was observed for gamithromycin in plasma and lung tissue (*R*^2^ ≥ 0.988 for C_max_ and AUC_last_). The T_1__/__2_ value was 0.67–0.83 h and 11.81–12.58 h for colistin and gamithromycin, respectively. In lung tissue, the AUC_last_ of colistin and gamithromycin was 5.22–12.20 mg⋅h/kg and 32.55–511.72 mg⋅h/kg, respectively. Correspondingly, the C_max_ was 3.37–5.54 mg/kg and 2.27–35.24 mg/kg, after 0.25–1 h.

**TABLE 4:** Pharmacokinetic parameters of colistin and gamithromycin in total plasma and lung tissue after subcutaneous administration in neutropenic lung-infected mice.

Antimicrobial	Dose (mg/kg)	Plasma				Lung				Lung/plasma^*a*^ AUC ratio
		T_max_ (h)	C_max_ (mg/L)	AUC_last_ (mg⋅h/L)	T_1/2_ (h)	T_max_ (h)	C_max_ (mg/kg)	AUC_last_ (mg⋅h/kg)	T_1/2_*** (h)	
Colistin	2.5	0.5	3.14	3.51	0.67	0.5	3.37	5.22	1.28	17.69
	5	0.75	6.33	10.52	0.83	0.75	5.54	12.20	1.72	13.80
Gamithromycin	1.5	0.75	0.11	0.26	11.89	1	2.27	32.55	23.84	145.3
	3	0.25	0.29	0.64	11.81	0.25	4.39	55.44	20.25	100.5
	6	0.5	0.59	1.44	12.32	0.75	7.43	122.12	22.40	98.42
	12	0.25	1.35	2.13	12.35	0.5	17.59	284.62	20.92	155.1
	24	0.5	2.52	5.06	12.58	0.75	35.24	511.72	24.18	117.4
	Mean ± SD	0.45 ± 0.21	NA	NA	12.19 ± 0.33	0.65 ± 0.29	NA	NA	22.58 ± 1.85	123.3 ± 25.82
Colistin/gamithromycin	2.5 (colistin)	0.5	3.33	3.88	0.61	0.5	2.94	5.10	1.16	15.65
	3 (gamithromycin)	0.25	0.30	0.60	10.25	0.5	4.24	56.77	21.92	109.80

*T_*max*_, time to maximum concentration of the drug in plasma; C_*max*_, maximum concentration of the drug in plasma; AUC_*last*_, the area under the concentration-time curve from 0 h to the last sampling time points; T_1__/__2_, half-life. ^*a*^The ratio of lung tissue AUC_*last*_ to free plasma AUC_*last*_. The protein binding percentage for colistin and gamithromycin in mouse plasma was 91.6% (Soon-Ee et al., 2015) and 13.83% (this study), respectively. ^∗∗∗^*P* < 0.001 for T_1__/__2_ of gamithromycin in the lung versus in the plasma.*

The results of plasma protein–binding experiments show that the protein binding extent of gamithromycin was 13.83% under the preparation method employed in this research. Based on these results and those of a previous report on colistin (unbound extent = 8.4%) (Soon-Ee et al., 2015), free plasma PK parameters were calculated, and the relevant ratio of AUC_last_ between plasma and lung tissue is shown in Table 4.

### *In vivo* Pharmacodynamics

**FIGURE 2:**
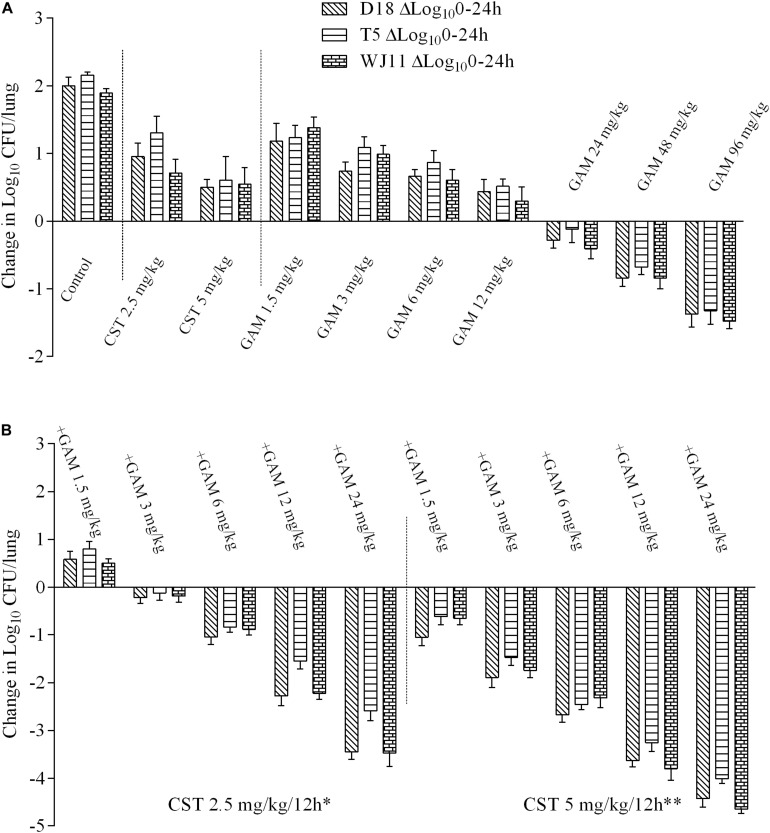
Log_10_ changes in mouse lung tissues (change in log_10_ CFU/lung) after 24 h of therapy with different dosing regimens against three *Pasteurella multocida* strains (D18, T5, and WJ11) in a mouse pneumonia model (*n* = 4). **(A)** *In vivo* pharmacodynamics of colistin sulfate at doses of 2.5 and 5 mg/kg and gamithromycin at doses of 1.5, 3, 6, 12, 24, 48, and 96 mg/kg alone against *P. multocida* strains. **(B)** *In vivo* pharmacodynamics of 2.5 and 5 mg/kg colistin sulfate in combination with gamithromycin at doses of 1.5, 3, 6, 12, and 24 mg/kg against *P. multocida* strains. A growth control without drug administration was included in all the tests. *P < 0.05 for antimicrobial activity in the combination treatments with 2.5 mg/kg colistin and gamithromycin versus gamithromycin monotherapy (1.5–24 mg/kg). **P < 0.01 for antimicrobial activity in the combination treatments with 5 mg/kg colistin and gamithromycin versus gamithromycin monotherapy (1.5–24 mg/kg).

Only an injection of more than 12 mg/kg gamithromycin into lung-infected mice displayed inhibitory activity with a bacterial burden decrease of 0.11–1.48 log_10_ CFU/lung, and none of the other monotherapy groups exhibited inhibition of bacterial growth (Figure 2A). No obvious differences were observed in the *in vivo* PD values with 2.5 or 5 mg/kg colistin for all three isolates, and failure to inhibit growth of bacteria was observed in all cases. As expected, a sequential decrease in bacterial burden was observed with increasing supplementation of gamithromycin to colistin. A bacterial eradication effect was evident as a reduction from 3.26 to 3.80-log_10_ CFU/lung and 4.01 to 4.65-log_10_ CFU/lung at 5 mg/kg colistin plus 12 and 24 mg/kg gamithromycin, respectively (Figure 2B). As above, 2.5 mg/kg colistin-based combinations with gamithromycin at 24 mg/kg also achieved >3 log_10_ kill activity except for strain T5. As observed for time-kill and MICs tests, the *in vivo* PD results showed no obvious differences among the three isolates except for 2.5 mg/kg colistin with 12 and 24 mg/kg gamithromycin against the T5 isolate.

### PK/PD Relationship Analysis

**FIGURE 3:**
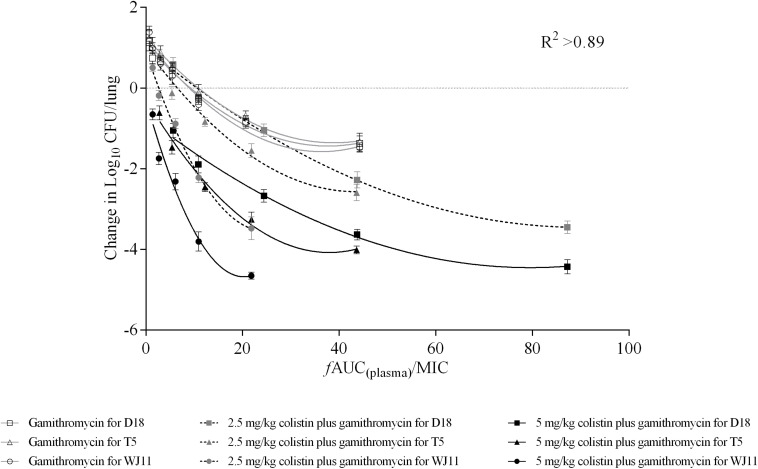
Correlations between plasma AUC/MIC ratios and *in vivo* pharmacodynamics (changes in log_10_ CFU/lung) after 24 h of monotherapy or combined therapy against *Pasteurella multocida* strains D18, T5, and WJ11. Each symbol represents the mean efficacy value (*n* = 4). Data points below the horizontal broken line represent bactericidal activity, and points above the line represent growth. *R*^2^ represents the coefficient of determination.

Integration of PK and PD data is important for clinical drug administration (Toutain et al., 2002). Herein, PK values in plasma were used to calculate the PK/PD index, which was integrated with changes in bacteria log_10_ CFU/lung. Antimicrobials that achieve significant PAE values are generally described as concentration-dependent (Jacobs, 2004). Thus, we explored the relationship between AUC_(__0__–__24 *h)*_/MIC for gamithromycin in plasma and the antimicrobial effect in the pneumonia model mice. A correlation with AUC_(__0__–__24 *h)*_/MIC > 0.89 indicated that this index may be suitable for predicting therapeutic outcomes (Figure 3). Using the dose-AUC/MIC reduction formula: Dose_(target)_ = Dose_(test)_ × AUC/MIC_(target)_/AUC/MIC_(test)_ (Shan et al., 2014), the target doses required to reach the target effect were calculated and are listed in Table 5 (AUC/MIC in Supplementary Table S1). The average doses required to reach stasis and a 1 × log_10_ kill effect for gamithromycin monotherapy were 18.43 ± 3.10 mg/kg and 67.11 ± 6.01 mg/kg, respectively. For 2.5 mg/kg colistin-based combinations, the required dose was reduced by 6- to 10-fold; average values of 2.79 and 5.35 mg/kg gamithromycin achieved the same effect. Moreover, doses required to reach 2 × and 3 × log_10_ kill effect were only 1/5 and 2/5 of the dose required to reach stasis following monotherapy, in which 5 mg/kg colistin was supplemented with gamithromycin.

**TABLE 5:** Gamithromycin doses required to reach stasis, 1 log_10_ kill, 2 log_10_ kill, and 3 log_10_ kill of *Pasteurella multocida* isolates in the lungs of mice for gamithromycin monotherapy and double therapy with colistin at doses of 2.5 and 5 mg/kg twice a day (*n* = 4).

Strain	Monotherapy (mg/kg)		Double therapy (mg/kg)						
	
	Gamithromycin		2.5 mg/kg colistin plus gamithromycin			5 mg/kg colistin plus gamithromycin			
	
	Stasis	1-log_10_ kill	Stasis*	1-log_10_ kill**	2-log_10_ kill	Stasis**	1-log_10_ kill**	2-log_10_ kill	3-log_10_ kill
D18	17.99	65.32	2.64	4.83	13.55	0.76	1.56	2.90	5.66
T5	21.73	73.80	2.94	5.82	21.88	0.99	2.08	3.96	11.60
WJ11	15.58	62.19	2.78	5.37	15.27	0.99	1.93	3.73	6.87
Mean ± SD	18.43 ± 3.10	67.11 ± 6.01	2.79 ± 0.15	5.35 ± 0.51	16.90 ± 4.40	0.92 ± 0.13	1.84 ± 0.26	3.67 ± 0.54	7.46 ± 1.81

*P < 0.05 for gamithromycin doses required to reach the same antimicrobial effect with double therapy versus gamithromycin monotherapy. **P < 0.01 for gamithromycin doses required to reach the same antimicrobial effect with double -therapy versus gamithromycin monotherapy.

## Discussion

Herein, five R isolates (D18, T5, XX12, PmP2, and PmP17) and four S isolates (CVCC444, WJ11, WW18, and WN17) were subject to MIC tests. Interestingly, synergistic and additive antimicrobial effects were observed against R and S isolates, respectively, when colistin was combined with gamithromycin. With regard to the mechanism of action, colistin may have damaged the outer membrane of *P. multocida*, thereby increasing the content of gamithromycin in the cytoplasm. To further investigate the potential mechanism of the combined activity of colistin and gamithromycin against *P. multocida* strains, changes in the transcriptome and protein content of *P. multocida* following treatment with drugs should be examined in the future.

Even though the MIC of colistin against WJ11 was 32-fold lower than that against isolate D18 or T5, each drug tended to reach the same concentration in combination treatments for both R and S isolates. Similarly, the rate and extent of killing for colistin and gamithromycin in combination did not differ between isolates D18 or T5 and WJ11 based on time-kill tests. Additionally, the killing effect was relatively insensitive to increases in the concentrations of colistin when the effect reached a certain level. The MPC results showed that the first-step resistant mutation ability of *P. multocida* in response to colistin was stronger than that for gamithromycin. It is worth noting that, although the MIC of colistin against WJ11 was 1 mg/L, the MPC was as high as 64 mg/L. This indicates that monotherapy with colistin is more likely to induce the development of drug-resistant mutants. Surprisingly, the combination of colistin and gamithromycin reduced the MPC of colistin considerably by 64-fold and closed the mutant selection window of colistin. This indicates that combination therapy is more reasonable and feasible. The *in vitro* PAE results also confirmed the combination of colistin and gamithromycin produced a more profound and lasting bactericidal activity *in vitro* even at lower concentrations of each drug. PAE should not be ignored from a therapeutic perspective.

The main PK characteristics of macrolides are good lung tissue permeability and low plasma content (Cazzola et al., 2002). Unsurprisingly, gamithromycin adhered to these characteristics in this study; the ratio of lung tissue AUC_last_ to free plasma AUC_last_ averaged 123.3, and there was no obvious interaction between PK indices for colistin and gamithromycin. The dose recommended by the EMA for cattle and swine for SC administration of gamithromycin is 6 mg/kg, which is associated with an AUC_last_ of 7.82 (Steeve et al., 2011) and 3.48 mg⋅h/L (Wyns et al., 2014). These values are similar to those for SC administration of 24 mg/kg gamithromycin in mice, as observed in this study. The faster removal of drugs in mice might explain this apparent discrepancy in those dose-specific effects, as the administration of a dose less than 24 mg/kg every 12 h failed to control the development of pneumonia. Meanwhile, single doses of 2.5 and 5 mg/kg colistin treatment every 12 h were also confirmed to be ineffective for lung-infected mice in the present study. Previous research showed the absence of colistin in lung after intravenous administration because of high colistin tissue binding (Lu et al., 2010). However, we found colistin concentrations in lung were comparable with those in plasma. Increased antimicrobial activity was observed by supplementation of colistin with gamithromycin in pneumonia model mice, and this is indicative of the potential efficacy of colistin against pneumonia. The influence of gamithromycin dose-time-fractionation on its *in vivo* antimicrobial effect was investigated in a preliminary experiment, which demonstrated that, when the total doses were unchanged, a double-dosing regimen every 24 h resulted in more positive treatment results than a single-dosing regimen every 24 h. According to the *in vivo* antimicrobial effects, effective treatment required ≥24 mg/kg gamithromycin every 12 h, equivalent to the efficacy produced by 2.5 mg/kg colistin-based combination with 3–6 mg/kg gamithromycin. An obvious bactericidal effect (>3-log_10_ reduction in colony count) was achieved by 2.5 mg/kg colistin plus 24 mg/kg gamithromycin. Furthermore, even greater killing of bacteria (>4 × log_10_ decrease in bacterial burden) could be achieved by combination therapy with 5 mg/kg colistin and 12 mg/kg gamithromycin.

Whether the traditional PK/PD concept is suitable for macrolides is controversial due to lower plasma and serum concentrations (Toutain et al., 2016). Infected lung tissues are the therapeutic targets of macrolides; hence, concentrations in the epithelial lining fluid are considered the most appropriate correlates with therapeutic efficacy. However, existing technologies and methods, such as bronchoalveolar lavage (BAL), fail to measure exact drug concentrations due to procedural errors that cannot be ignored. Even though there was no direct relationship with changes in bacterial burden in lung tissue, the approximate free drug levels in the plasma based on the MICs are considered to be the best estimates (Sungmin and Schentag, 2008). In this study, there was good compliance between the therapeutic regimens and antimicrobial efficacy based on the relationship between PK and PD in plasma. AUC_(__0__–__24 *h)*_/MIC was considered to be the best indicator of therapeutic outcomes, and the target values required to achieve the same antimicrobial activity for combination therapy were 6- to 35-fold lower than those for monotherapy. Subsequently, an equivalent dose based on a comparison of the body surface area of mice and other animals could be calculated to predict the appropriate clinical target dose (Wojcikowski and Gobe, 2014). A lower dose than the clinically recommended one might be more achievable with colistin-based combination therapy than with colistin or gamithromycin monotherapy in target animals. Furthermore, the difference in concentration based on the difference in the permeability of drugs in lung tissue between mice and target population could be responsible for the differences in PK and PD between target animals. Along with the clearance rate, the free fraction of drugs, and their bioavailability, dose optimization should be examined and verified based on the penetration rate of colistin and gamithromycin in the lung tissue of target animals (Toutain et al., 2007).

The newfound macrolide efflux gene *mef(B)* (Liu et al., 2009) was identified in several *P. multocida* stains with gamithromycin MICs of 0.5 mg/L in this study. This finding indicates that gamithromycin resistance might not be conferred by the *mef*(B) gene. Despite this, gamithromycin-resistant strains generated by the active efflux of drugs associated with the *erm*(42) and *msr*(E) – *mph*(E) genes have not been obtained so far, and the addition of colistin to gamithromycin may be effective against resistant strains via a mechanism that potentially involves an efflux pump. Future studies should examine whether the enhanced antimicrobial activity is reproducible in gamithromycin-resistant strains.

Herein, the increased antimicrobial activities of colistin-based combinations with gamithromycin against *P. multocida* strains *in vitro* were determined and verified by adopting a mouse pneumonic model. It is interesting to note that the concentrations of the combination of colistin and gamithromycin tended to reach similar levels against different *P. multocida* strains even though there was a considerable difference in the colistin sensitivity of these strains. Additionally, the administration regimen was optimized by assessing the relationship between gamithromycin PK data in plasma and the antimicrobial efficacy in lung tissue. The findings indicate that combining colistin with gamithromycin holds promise for treating pneumonia caused by *P. multocida* strains.

## Data Availability Statement

The datasets generated for this study are available on request to the corresponding author.

## Ethics Statement

The animal study was reviewed and approved by the Committee on the Ethics of Animals of South China Agricultural University.

## Author Contributions

YL carried out the main experiments, data analysis, and wrote the manuscript. MX, JZ, and HL took part in the animal study. TX and LW participated in the isolation and identification of bacteria experiments. HD participated in the data analysis. BF conceived and designed the experiments. All authors contributed to the article and approved the submitted version.

## Conflict of Interest

The authors declare that the research was conducted in the absence of any commercial or financial relationships that could be construed as a potential conflict of interest.
