# Supplementary material for: Increased Antimicrobial Activity of Colistin in Combination With Gamithromycin Against Pasteurella multocida in a Neutropenic Murine Lung Infection Model
Source: Front Microbiol. 2020 Sep 22;11:511356. doi: 10.3389/fmicb.2020.511356 (PMC7536268; doi:10.3389/fmicb.2020.511356)
Supplement: Supplementary file 1 [file Table_1.doc]

Supplementary Materials for

**Increased Antimicrobial Activity of Colistin in Combination with Gamithromycin Against** ***Pasteurella multocida* in a Neutropenic Murine Lung Infection Model**

Yanqin Li^1,2^, Mengjuan Xie^1,2^, Junwen Zhou^1,2^, Hao Lin^1,2^, Tianan Xiao^3^, Liqin Wu^3^, Huanzhong Ding^1,2^, Binghu Fang^1,2*^

**The file includes:**

**Supporting TABLE S1** Gamithromycin AUC/MIC required to reach stasis, 1 log_10_ kill, 2 log_10_ kill, and 3 log_10_ kill of *Pasteurella multocida* isolates in lungs of mice for gamithromycin monotherapy and double therapy with colistin at doses of 2.5 and 5 mg/kg twice a day (n = 4).

TABLE S1 Gamithromycin AUC/MIC required to reach stasis, 1 log10 kill, 2 log10 kill, and 3 log10 kill of *Pasteurella multocida* isolates in lungs of mice for gamithromycin monotherapy and double therapy with colistin at doses of 2.5 and 5 mg/kg twice a day (n = 4).

| Strain | Monotherapy (AUC/MIC) | |  | Double therapy (AUC/MIC) | | | | | | | |
| --- | --- | --- | --- | --- | --- | --- | --- | --- | --- | --- | --- |
|  | Gamithromycin | |  | 2.5 mg/kg colistin plus gamithromycin | | |  | 5 mg/kg colistin plus gamithromycin | | | |
|  | Stasis | 1-log_10_ kill |  | Stasis* | 1-log_10_ kill** | 2-log_10_ kill |  | Stasis** | 1-log_10_ kill** | 2-log_10_ kill | 3-log_10_ kill |
| D18 | 10.33 | 36.74 |  | 12.90 | 28.36 | 56.44 |  | 3.47 | 7.14 | 14.18 | 33.26 |
| T5 | 12.47 | 41.51 |  | 7.18 | 17.07 | 50.24 |  | 2.27 | 4.75 | 9.67 | 24.16 |
| WJ11 | 8.11 | 34.98 |  | 3.39 | 7.87 | 15.90 |  | 1.13 | 2.24 | 4.34 | 9.82 |

*P < 0.05 for gamithromycin AUC/MIC required to reach the same antimicrobial effect with double therapy versus gamithromycin monotherapy. **P < 0.01 for gamithromycin AUC/MIC required to reach the same antimicrobial effect with double therapy versus gamithromycin monotherapy.
